# Supplementary figures and images for: B cell subpopulations and their role in the pathogenesis of primary Sjögren’s syndrome: insights from single-cell RNA sequencing
Source: Front Immunol. 2025 Oct 16;16:1665086. doi: 10.3389/fimmu.2025.1665086 (PMC12571843; doi:10.3389/fimmu.2025.1665086)

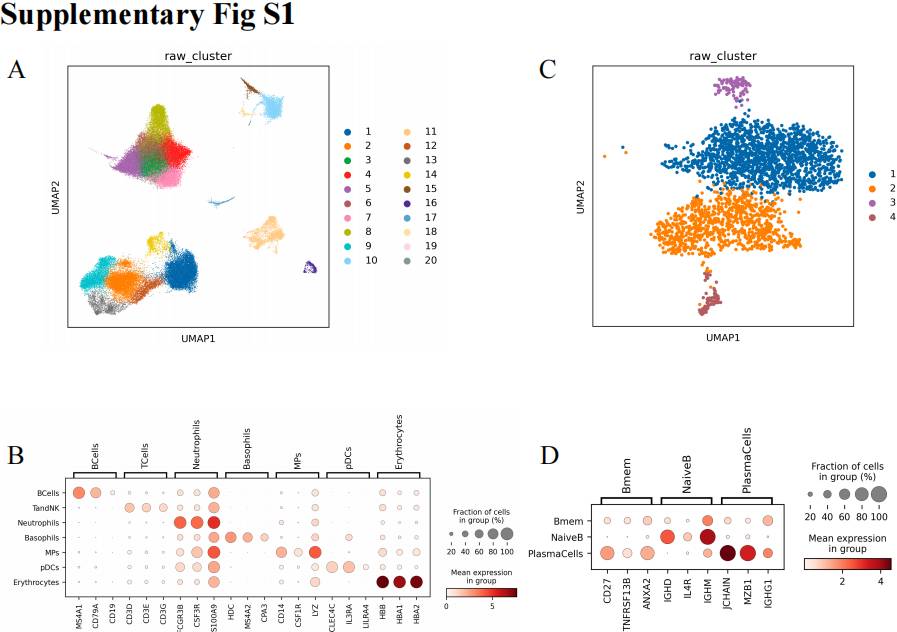

Supplement: Supplementary file 1 [file Image1.jpeg]

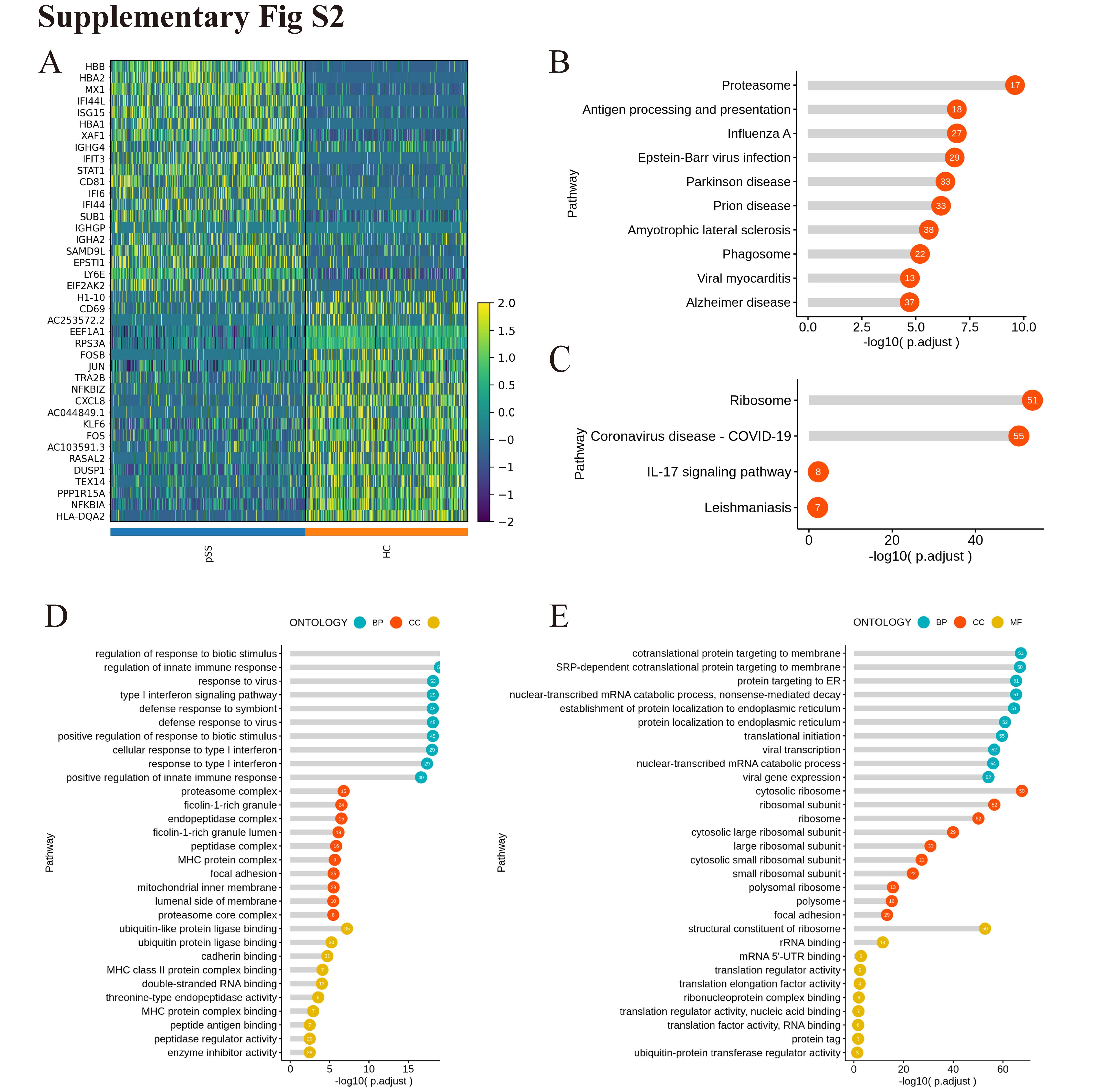

Supplement: Supplementary file 2 [file Image2.jpeg]

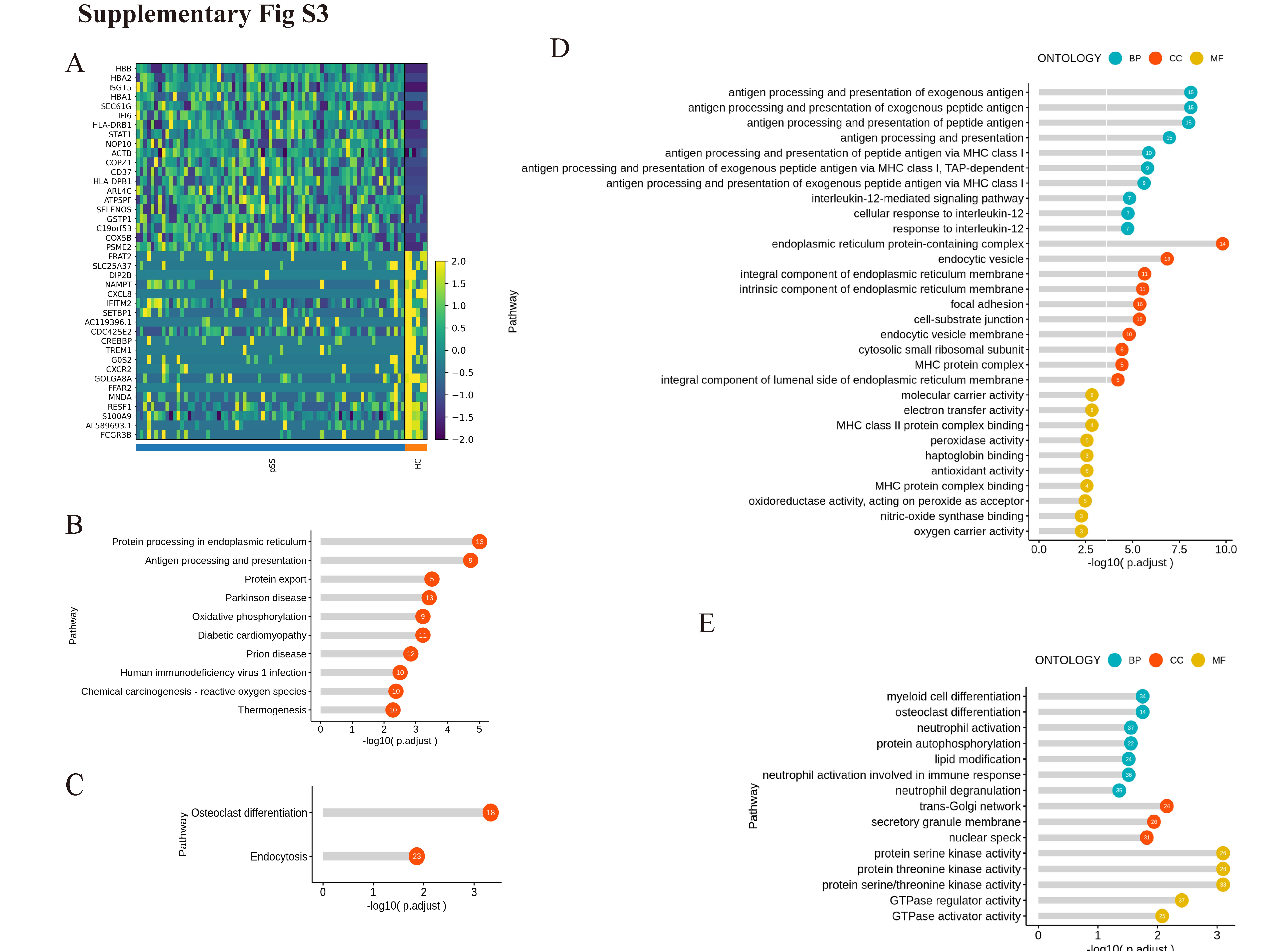

Supplement: Supplementary file 3 [file Image3.jpeg]

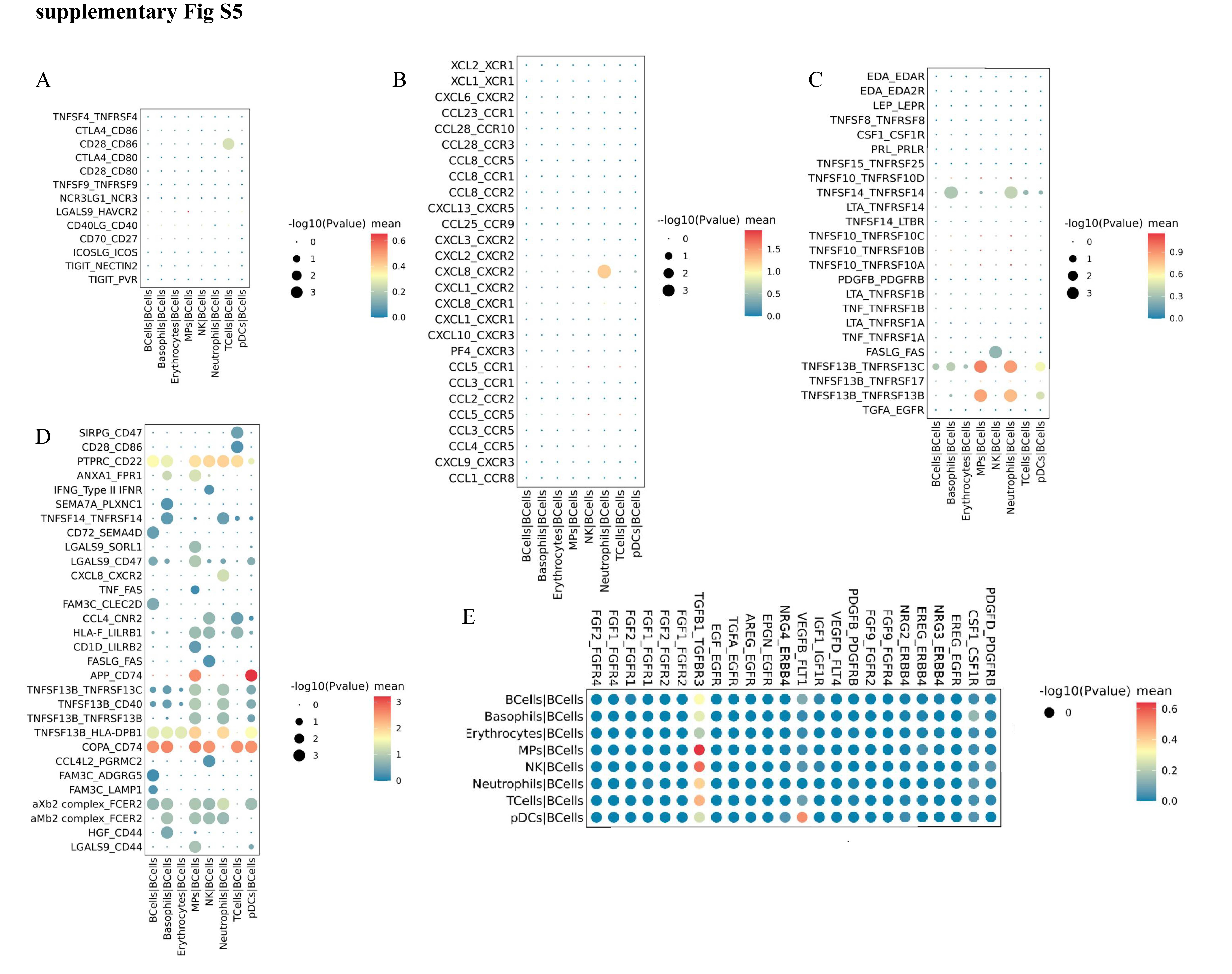

Supplement: Supplementary file 5 [file Image5.jpeg]
