# Supplementary material for: B cell subpopulations and their role in the pathogenesis of primary Sjögren’s syndrome: insights from single-cell RNA sequencing
Source: Front Immunol. 2025 Oct 16;16:1665086. doi: 10.3389/fimmu.2025.1665086 (PMC12571843; doi:10.3389/fimmu.2025.1665086)
Supplement: Supplementary file 6 [file Table1.docx]

**Supplementary Table1**

**Table of cell population counts across 3 pSS and 3 HC samples**

| cluster | HC01 | HC02 | HC03 | pSS01 | pSS02 | pSS03 |
| --- | --- | --- | --- | --- | --- | --- |
| BCells | 530 | 330 | 309 | 803 | 505 | 461 |
| NK | 464 | 184 | 535 | 1150 | 279 | 772 |
| TCells | 3120 | 3032 | 1587 | 6544 | 2929 | 2605 |
| Neutrophils | 5506 | 5377 | 9353 | 598 | 7850 | 5235 |
| Basophils | 21 | 45 | 111 | 101 | 61 | 168 |
| MPs | 122 | 200 | 567 | 1648 | 899 | 1208 |
| pDCs | 6 | 7 | 6 | 5 | 9 | 9 |
| Erythrocytes | 6 | 21 | 10 | 293 | 73 | 16 |
| Bmem | 154 | 91 | 150 | 304 | 76 | 95 |
| NaiveB | 322 | 207 | 118 | 328 | 347 | 266 |
| PlasmaCells | 0 | 2 | 4 | 60 | 3 | 10 |

**Supplementary Table 2**

**Table of clonal frequency distribution in 3 pSS samples and 3 HC samples3**

|  | sample | celltype | Medium | Single | Large |
| --- | --- | --- | --- | --- | --- |
| 1 | HC01 | Bmem | 10 | 114 | 0 |
| 2 | HC01 | NaiveB | 8 | 256 | 0 |
| 3 | HC02 | Bmem | 1 | 66 | 0 |
| 4 | HC02 | NaiveB | 1 | 139 | 0 |
| 5 | HC03 | Bmem | 3 | 57 | 0 |
| 6 | HC03 | NaiveB | 0 | 55 | 0 |
| 7 | HC03 | PlasmaCells | 1 | 2 | 0 |
| 8 | pSS01 | Bmem | 24 | 148 | 0 |
| 9 | pSS01 | NaiveB | 13 | 222 | 0 |
| 10 | pSS01 | PlasmaCells | 14 | 45 | 0 |
| 11 | pSS02 | Bmem | 0 | 11 | 0 |
| 12 | pSS02 | NaiveB | 0 | 117 | 0 |
| 13 | pSS02 | PlasmaCells | 0 | 1 | 0 |
| 14 | pSS03 | Bmem | 3 | 53 | 0 |
| 15 | pSS03 | NaiveB | 3 | 175 | 0 |
| 16 | pSS03 | PlasmaCells | 0 | 10 | 0 |
| 17 | HC01 | alltype | 18 | 370 | 0 |
| 18 | HC02 | alltype | 2 | 205 | 0 |
| 19 | HC03 | alltype | 4 | 114 | 0 |
| 20 | pSS01 | alltype | 51 | 415 | 0 |
| 21 | pSS02 | alltype | 0 | 129 | 0 |
| 22 | pSS03 | alltype | 6 | 238 | 0 |
| 23 | allsample | Bmem | 41 | 449 | 0 |
| 24 | allsample | NaiveB | 25 | 964 | 0 |
| 25 | allsample | PlasmaCells | 15 | 58 | 0 |
| 26 | allsample | alltype | 81 | 1471 | 0 |

Note: Single represents unique clonotypes; Medium denotes clonotypes with frequency >1 and ≤10; Large indicates clonotypes with frequency >10.

**Supplementary Fig S1 | Unsupervised clustering and marker gene expression of PBMCs and B cells**

(A) Unsupervised clustering of PBMCs (UMAP visualization).

(B) Dot plot of canonical marker genes across major PBMC clusters. Bubble size represents the proportion of cells expressing a given gene, while bubble color indicates the average expression level.

(C) Unsupervised clustering of B cells (UMAP visualization).

(D) Dot plot of representative marker genes in B cell clusters. Bubble size represents the percentage of cells expressing the marker, and bubble color reflects the expression intensity.

**Supplementary Fig S2 | Inter-group Comparison and Functional Analysis of Differential Genes in the Naive B Subset**

(A) Heatmap of top 20 differential gene expression comparisons between groups in

the Naive B subset.

(B) KEGG pathway analysis of upregulated genes in the Naive B subset of the pSS

group.

(C) KEGG pathway analysis of downregulated genes in the Naive B subset of the pSS

group.

(D) GO term analysis of upregulated genes in the Naive B subset of the pSS group.

(E) GO term analysis of downregulated genes in the Naive B subset of the pSS group.

**Supplementary Fig S3 | Inter-group Comparison and Functional Analysis of Differential Genes in the Plasma Cells Subset**

(A) Heatmap of top 20 differential gene expression comparisons between groups in the Plasma Cells subset.

(B) KEGG pathway analysis of upregulated genes in the Plasma Cells subset of the pSS group.

(C) KEGG pathway analysis of downregulated genes in the Plasma Cells subset of the pSS group.

(D) GO term analysis of upregulated genes in the Plasma Cells subset of the pSS group.

(E) GO term analysis of downregulated genes in the Plasma Cells subset of the pSS group.

**Supplementary Fig S4| Interaction pairs between B cells as ligand cells and other cell types in the pSS group**

(A) Bubble plot of immune checkpoint (checkpoint) interaction pairs between B cells and other cell types.

(B) Bubble plot of chemokine (chemokine) interaction pairs between B cells and other cell types.

(C) Bubble plot of cytokine (cytokine) interaction pairs between B cells and other cell types.

(D) Bubble plot of growth factor (growth factor) interaction pairs between B cells and other cell types.

(E) Bubble plot of Rank30 interaction pairs between B cells as ligand cells and other cell types.

**Supplementary Fig S5| Interaction pairs between B cells as receptor cells and other cell types in the pSS group**

(A) Bubble plot of immune checkpoint (checkpoint) interaction pairs between B cells and other cell types.

(B) Bubble plot of chemokine (chemokine) interaction pairs between B cells and other cell types.

(C) Bubble plot of cytokine (cytokine) interaction pairs between B cells and other cell types.

(D) Bubble plot of growth factor (growth factor) interaction pairs between B cells and other cell types.

(E) Bubble plot of Rank30 interaction pairs between B cells as ligand cells and other cell types.
